# Seroprevalence of Maternal and Cord Antibodies Specific for Diphtheria, Tetanus, Pertussis, Measles, Mumps and Rubella in Shunyi, Beijing

**DOI:** 10.1038/s41598-018-31283-y

**Published:** 2018-08-29

**Authors:** Qing-hong Meng, Ying Liu, Jin-qian Yu, Li-jun Li, Wei Shi, Ying-jie Shen, Li Li, Shi-na Zhan, Fan Yang, Ya-juan Wang, Kai-hu Yao

**Affiliations:** 1 Beijing Key Laboratory of Pediatric Respiratory Infection Diseases, Key Laboratory of Major Diseases in Children, Ministry of Education, National Clinical Research Center for Respiratory Diseases, National Key Discipline of Pediatrics (Capital Medical University), Beijing Pediatric Research Institute, Beijing Children’s Hospital, Capital Medical University, National Center for Children’s Health, Beijing, 100045 China; 2 Department of Neonatology, Beijing Children’s Hospital, Capital Medical University, National Center for Children’s Health, Beijing, 100045 China; 3 Department of Neonatology, Shunyi Women and Children’s Hospital of Beijing Children’s Hospital, Beijing, 101300 China

## Abstract

Maternal antibodies contribute to the protection of young infants from infectious diseases during the early life. However, vaccinations for women of child-bearing age are not routine in China. Therefore, we investigated the level of protective immunity against vaccine preventable diseases in pregnant women and newborns in China. A total of 194 paired maternal and cord blood samples were collected in Beijing from 2016 to 2017. Antibodies specific for the antigens covered by diphtheria-tetanus-pertussis (DTP) and measles-mumps-rubella (MMR) vaccine were determined by ELISA (Euroimmun, Lübeck, Germany). The cut off value of ≥0.1 IU/ml (anti-diphtheria), >0.1 IU/ml (anti-tetanus), >40 IU/ml (anti-pertussis toxin), ≥200 IU/l (anti-measles), ≥45 RU/ml (anti-mumps) and ≥10 IU/ml (anti-rubella) were used to assess the percentage of newborns with protective IgG concentrations, respectively. The results revealed that 61.3%, 73.2%, 97.4%, 30.4%, 65.5% and 17.0% of newborns had no protection against diphtheria, tetanus, pertussis, measles, mumps and rubella. Only 1.0% and 23.7% of newborns had protection against all three components of DTP or MMR, respectively. The finding suggested that most of newborns were susceptible to diphtheria, tetanus, pertussis and mumps, almost one-third of this population had no immune protection against measles, and about one-sixth of them were under threat of rubella infection. These data supported the immunization program for DTP and MMR vaccine in women at child-bearing age.

## Introduction

Infant immunization has been used for prevention and treatment of many serious infectious diseases^1^. However, newborns are still at high risk because of an immature immune system, which is not capable of protecting them actively against vaccine preventable infections. Traditionally, the newborns and young infants could acquire indirect protection by maternal antibody, which was transferred through the placenta and lactation^2,3^.

Vaccination against pertussis and measles was introduced to China in 1960s^4,5^, however, a national immunization program first established until 1978. A routine immunization schedule that included 4 basic vaccines against 6 contagious diseases (tuberculosis, diphtheria, neonatal tetanus, whooping cough, poliomyelitis, and measles) was first established^6^. Hepatitis B vaccine was added into the routine immunization schedule in 2002. From 2007, a combined DTaP vaccine was introduced in Expanded Program on Immunization (EPI) of China, both DTwP and DTaP were used^7,8^. The first immunization of DTP is administrated in the third month after birth, which is followed other two dosages in the fourth and fifth month for the basic immunization. The children are inoculated again for the booster immunization at 18–24 months, another dose of DT vaccine are inoculated at 6 years. Meanwhile, the national immunization schedule was also expanded from 5 vaccines against 7 contagious diseases to 14 vaccines against 15 infectious diseases, mumps and rubella vaccine was introduced into the national immunization schedule at this opportunity^6^. Since 1985, the first immunization of MV was administrated at 9–12 month after birth, which was followed another dosage at 7 years^4^. From 2007, MV was replaced by a measles-rubella vaccine (MRV) at 8 months of age, followed by a MMR vaccine at 18–24 months of age^9^.

Even with childhood immunization, antibody levels decreased over time. Many infectious diseases remain the main public health concern. For example, pertussis resurgence has been reported in many developed countries^10^, such as the United States^11^, Spain^12^ and the United Kingdom^13^. Therefore, at present, vaccination of pregnant women or non-pregnant women of child-bearing age is recommended for protection of newborns. In the United States, the Advisory Committee on Immunization Practices (ACIP) recommended that pregnant women should receive 1 dose of tetanus and diphtheria toxoids and acellular pertussis vaccine (Tdap) during each pregnancy^14,15^. ACIP also recommended that non-pregnant women of child-bearing age without evidence of rubella immunity should receive 1 dose of measles-mumps-rubella (MMR)^14,15^.

In China, the epidemiological data showed that some diseases were also re-emerging. The numbers of pertussis case reported to China CDC increased obviously in recent years, from 1709 cases in 2013, increased to 5584 in 2016, and 10390 in 2017, respectively^16^. Several seroepidemiological studies even indicated that the incidence of pertussis was most likely underestimated in China. Resurgence of measles in recent years has also impeded the goal of China to eliminate measles. The number of measles case has remained at level more than 20000, the exact number reported to China CDC were 52628 in 2014, 42361 in 2015, and 24820 in 2016, respectively^16^. With the implementation of the two-child policy, a baby boom is coming in the next few years in China. However, no Tdap booster for adolescents or pregnant women is introduced, and MMR vaccinations of adolescents or women of child-bearing age are not routine, therefore, maternal antibody mainly depends on childhood immunizations and nature infections in China.

Here, we investigated antibodies specific for DTP and MMR vaccination in paired maternal and cord blood samples, in order to gain an insight into the immunity level against vaccine preventable diseases in pregnant women and newborns in China.

## Results

### Study participants

A total of 194 pregnant women in the Shunyi Women and Children’s Hospital of Beijing Children’s Hospital were enrolled in the study. The mean maternal age at the time of delivery was 28.6 years (21–39 years). The mean gestational age was 38.3 weeks (28.1–41.3 weeks). The distributions of female and male newborns were 49.5% and 50.5%, respectively.

### Seroprevalence of DTP and MMR vaccine-specific IgG in maternal and cord sera

**Figure 1:**
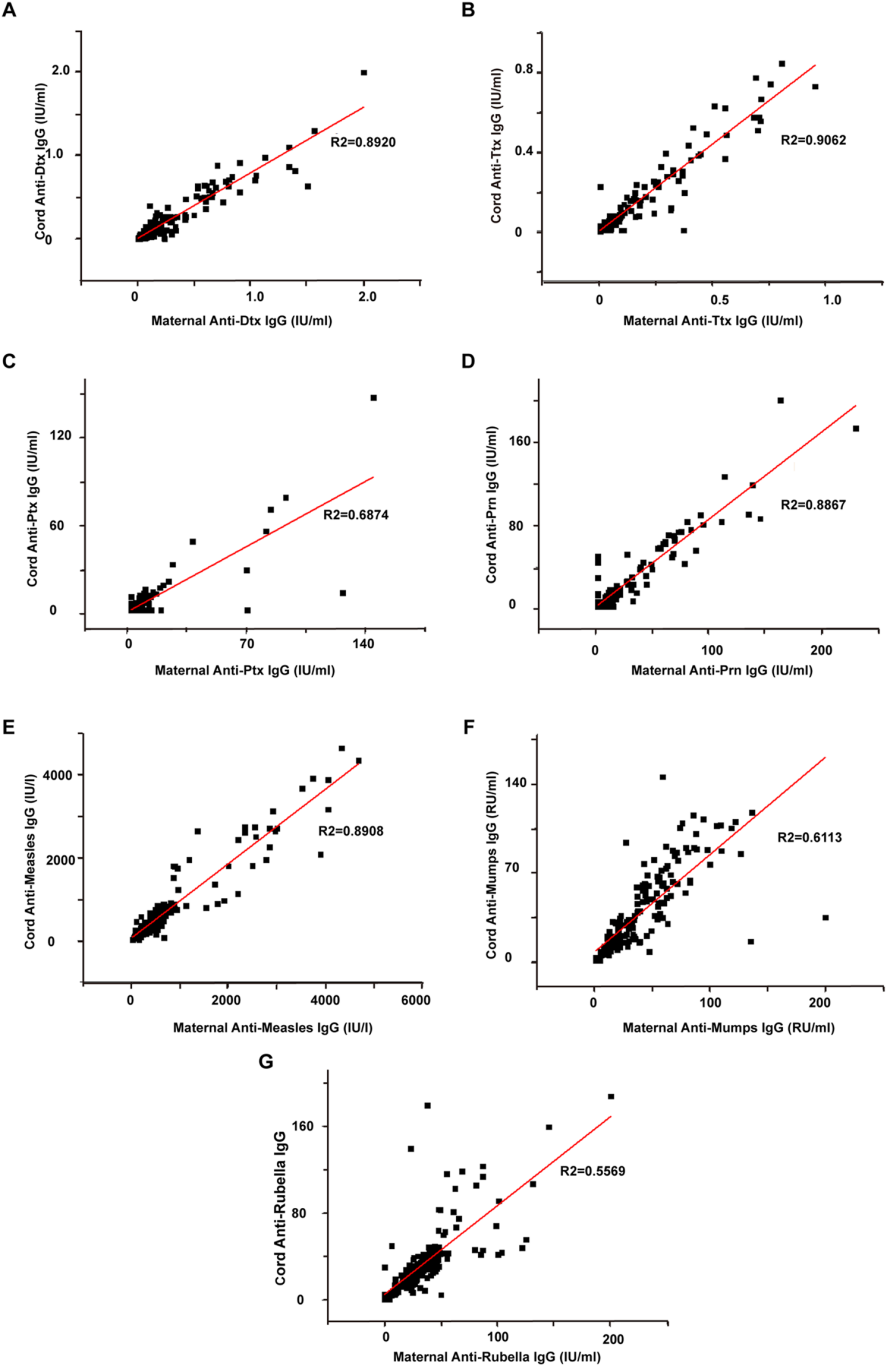
Individual maternal-to-cord antibody levels of anti-Dtx (**A**), anti-Ttx (**B**), anti-Ptx (**C**), anti-Prn (**D**), anti-measles (**E**), anti-mumps (**F**) and anti-rubella IgG (**G**).

The 25%, 50% and 75% percentiles for DTP and MMR vaccine-specific IgG in maternal and cord sera were summarized in Table 1. A strong correlation between maternal and cord serum antibody levels was found for anti-diphtheria (Dtx) (R^2^ = 0.8920), anti-tetanus (Ttx) (R^2^ = 0.9062), anti-pertussis toxin (Ptx) (R^2^ = 0.6874), anti-pertactin (Prn) (R^2^ = 0.8867), anti-measles (R^2^ = 0.8908), anti-mumps (R^2^ = 0.6113) and anti-rubella (R^2^ = 0.5569). Individual maternal and cord antibody concentrations to these antibodies were shown in Fig. 1.

**Table 1:** Concentrations of anti-Dtx, anti-Ttx, anti-Ptx, anti-Prn, anti-measles, anti-mumps and anti-rubella antibodies in maternal and cord sera.

Antibody	25% percentiles		50% percentiles		75% percentiles	
	Maternal	Cord	Maternal	Cord	Maternal	Cord
Anti-Dtx (IU/ml)	0.04	0.03	0.08	0.07	0.25	0.23
Anti-Ttx (IU/ml)	0.02	0.01	0.05	0.04	0.15	0.12
Anti-Ptx (IU/ml)	<5	<5	<5	<5	5.58	5.15
Anti-Prn (IU/ml)	<5	<5	<5	<5	18.79	20.29
Anti-Measles (IU/l)	178.02	179.04	411.97	377.40	681.72	747.10
Anti-Mumps (RU/ml)	13.81	12.82	29.02	25.01	56.08	58.44
Anti-Rubella (IU/ml)	19.12	15.38	29.96	28.15	43.58	40.36

Note. Dtx, diphtheria; Ttx, tetanus; Ptx, pertussis toxin; Prn, pertactin.

### DTP vaccine-specific antibody in maternal and cord sera

The distributions of DTP vaccine-specific IgG in maternal and cord sera were presented in Tables 2–4. In the 194 paired maternal and cord sera, only 8 newborns had anti-Dtx IgG that was below the lower limit of detection (0.01 IU/ml). More than half of the mothers (55.7%, 95% CI: 48.6–62.5%) have no immune protection (<0.1 IU/ml). Nearly 40% of mothers (39.7%, 95% CI: 33.1–46.7%) showed only basic protection (0.1–1.0 IU/ml). Only 4.6% (95% CI: 2.5–8.6%) of mothers have long term protection (>1.0 IU/ml) (Table 2).

**Table 2:** The distribution of anti-Dtx antibodies in maternal and cord sera.

Anti-Dtx (IU/ml)	Prevalence% (95% CI)	
	Cord	Maternal
<0.01	4.1 (2.1–7.9)	0 (0–1.9)
0.01–<0.1	57.2 (50.2–64.0)	55.7 (48.6–62.5)
0.1–1.0	37.1 (30.6–44.1)	39.7 (33.1–46.7)
>1.0–1.5	1.0 (0.3–3.7)	3.1 (1.4–6.6)
>1.5–2.0	0 (0–1.9)	1.5 (0.5–4.4)
>2.0	0.5 (0.1–2.9)	0 (0–1.9)

**Table 3:** The distribution of anti-Ttx antibodies in maternal and cord sera.

Anti-Ttx (IU/ml)	Prevalence% (95% CI)	
	Cord	Maternal
<0.01	24.2 (18.7–30.7)	18.6 (13.7–24.6)
0.01–0.1	49.0 (42.0–56.0)	52.6 (45.6–59.5)
>0.1–0.5	20.6 (15.5–26.9)	22.2 (16.9–28.5)
>0.5–1.0	6.2 (3.6–10.5)	6.7 (4.0–11.1)

**Table 4:** The distribution of anti-Ptx and anti-Prn antibodies in maternal and cord sera.

Antiboy(IU/ml)	Prevalence% (95% CI)			
	Anti-Ptx		Anti-Prn	
	Cord	Maternal	Cord	Maternal
<5	74.7 (68.2–80.3)	70.0 (63.0–76.0)	51.5 (44.6–58.5)	51.5 (44.6–58.5)
5–<40	22.7 (17.4–29.1)	26.3 (20.6–32.9)	31.4 (25.3–38.3)	32.0 (25.8–38.8)
40–<100	2.1 (0.8–5.2)	2.6 (1.1–5.9)	14.9 (10.6–20.6)	12.9 (8.9–18.3)
≥100	0.5 (0.1–2.9)	1.0 (0.3–3.7)	2.1 (0.8–5.2)	3.6 (1.8–7.3)

For anti-Ttx IgG, 36 mothers and 47 newborns had anti-Ttx IgG below the lower limit of detection (0.01 IU/ml). About 70% of the mothers (71.1%, 95% CI: 66.4–77.1%) have no immune protection (≤0.1 IU/ml), and 20% (22.2%, 95% CI: 16.9–28.5%) of mothers showed only basic protection (>0.1–0.5 IU/ml). Only 6.7% (95% CI: 4.0–11.1%) of mothers have long term protection (>0.5–1.0 IU/ml) (Table 3).

In the 194 paired maternal and cord sera, anti-Ptx IgG in 136 (70.1%, 95% CI: 63.6–76.1%) mothers and 145 (74.8%, 95% CI: 68.2–80.3%) newborns were below the lower limit of detection (5 IU/ml). Even with detectable anti-Ptx antibodies, the majority of mothers (81.0%, 47/58) and newborns (83.7%, 41/49) had antibody level of 5–20 IU/ml. Anti-Prn IgG below the lower limit of detection (5 IU/ml) occurred in 100 (51.5%, 95% CI: 44.6–58.5%) mothers and 100 (51.5%, 95% CI: 44.6–58.5%) newborns (Table 4).

### MMR vaccine-specific antibody in maternal and cord sera

The distributions of MMR vaccine-specific IgG in maternal and cord sera were presented in Table 5. In 194 paired maternal and cord sera, only 2, 8 and 11 mothers had anti-measles, anti-mumps and anti-rubella IgG below the lower limit of detection.

**Table 5:** The distribution of anti-measles, anti-mumps and anti-rubella antibodies in maternal and cord sera.

Group	Prevalence% (95% CI)					
	Anti-measles		Anti-mumps		Anti-rubella	
	Cord	Maternal	Cord	Maternal	Cord	Maternal
Negative	30.4 (24.4–37.2)	29.9 (23.9–36.7)	34.0 (27.7–40.9)	29.9 (23.9–36.7)	14.4 (10.2–20.1)	11.3 (7.6–16.6)
Equivocal	10.3 (6.8–15.4)	9.8 (6.4–14.8)	13.9 (9.7–19.5)	14.9 (10.6–20.6)	2.6 (1.1–5.9)	5.7 (3.2–9.9)
Positive	59.3 (52.2–65.9)	60.3 (53.3–66.9)	52.1 (45.1–59.0)	55.2 (48.1–62.0)	83.0 (77.1–87.6)	83.0 (77.1–87.6)

In the tested cases, 59.3% (95% CI: 52.2–65.9%)/60.3% (95% CI: 53.3–66.9%), 52.1% (95% CI: 45.1–59.0%)/55.2% (95% CI: 48.1–62.0%), and 83.0% (95% CI: 77.1–87.6%)/83.0% (95% CI: 77.1–87.6%) of newborns/mothers were positive for anti-measles, anti-mumps and anti-rubella IgG, respectively.

### Protective antibody levels in cord sera

Newborns with protective antibody were presented in Table 6. Protective concentrations of anti-Dtx IgG (≥0.1 IU/ml), anti-Ttx IgG (>0. 1 IU/ml), anti-Ptx IgG (>40 IU/ml)^17^, anti-measles (≥200 IU/l)^18,19^, anti-mumps IgG (≥45 RU/ml)^18,19^ and anti-rubella (≥10 IU/ml)^18–20^ were determined in 38.7% (95% CI: 32.1–45.7%), 26.8% (95% CI: 21.1–33.4%), 2.6% (95% CI: 1.1–5.9%), 69.6% (95% CI: 62.8–75.6%), 34.5% (95% CI: 28.2–41.5%) and 83.0% (95% CI: 77.1–87.6%) of cord sera, respectively.

**Table 6:** Prevalence of protective DTP and MMR associated antibody in cord sera with maternal demographic characters.

Maternal variable	N	Protective % (95% CI)					
		Anti-Dtx	Anti-Ttx	Anti-Ptx	Anti-measles	Anti-mumps	Anti-rubella
		(≥0.1 IU/ml)	(>0. 1 IU/ml)	(>40 IU/ml)	(≥200 IU/l)	(≥45 RU/ml)	(≥10 IU/ml)
**Total**	194	38.7 (32.1–45.2)	26.8 (21.1–33.4)	2.6 (11.1–59.0)	69.6 (62.8–75.6)	34.5 (28.2–41.5)	83.0 (77.1–87.6)
**Age**							
≤26 y	60	55.0 (42.5–66.9)	36.7 (25.6–49.3)	1.7 (0.3–8.9)	75.0 (62.8–84.2)	43.3 (31.6–55.9)	81.7 (70.1–89.4)
>26 y	134	31.3 (24.1–39.6)	22.4 (16.2–30.2)	3.0 (1.2–7.4)	67.2 (58.8–74.5)	30.6 (23.4–38.8)	83.6 (76.4–88.9)
P		0.0018	0.0380	0.5922	0.2728	0.0846	0.7428
**Gravidity**							
1	92	42.4 (32.8–52.6)	32.6 (23.9–42.7)	4.3 (1.7–10.6)	69.6 (59.5–78.0)	35.9 (26.8–46.1)	79.3 (70.0–86.4)
>1	102	35.3 (26.7–45.0)	21.6 (14.7–30.5)	1.0 (0.2–5.3)	69.6 (60.1–77.7)	33.3 (24.7–42.9)	86.3 (78.3–91.6)
P		0.3108	0.0830	0.1394	0.9949	0.7017	0.1998
**Parity**							
1	142	43.0 (35.1–51.2)	30.3 (23.3–38.3)	2.8 (1.1–7.0)	70.4 (62.5–77.3)	35.9 (28.5–44.1)	82.4 (75.3–87.8)
>1	52	26.9 (16.8–40.2)	17.3 (9.4–29.7)	1.9 (0.3–10.1)	67.3 (53.8–78.5)	30.8 (20.0–44.3)	84.6 (72.5–92.0)
P		0.0422	0.0708	0.7278	0.6761	0.3573	0.7153
**Birth place**							
Beijing	96	54.2 (44.2–63.8)	38.5 (29.4–48.5)	1.0 (0.2–5.7)	66.7 (56.8–75.3)	36.5 (27.5–46.4)	80.2 (71.1–86.9)
Other city	98	23.5 (16.2–32.8)	15.3 (9.5–23.7)	4.1 (1.6–10.0)	72.4 (62.9–80.3)	32.7 (24.2–42.4)	85.7 (77.4–91.3)
P		<0.0001	0.0003	0.1816	0.3814	0.5773	0.3075
**Residence place**							
Urban	71	32.4 (22.7–43.9)	23.9 (15.5–35.0)	1.4 (0.2–7.0)	66.2 (54.6–76.1)	29.6 (20.2–41.0)	85.9 (76.0–92.2)
Rural	123	42.3 (33.9–51.5)	28.5 (21.2–37.0)	3.3 (1.3–8.1)	71.5 (63.0–78.8)	37.4 (29.4–46.2)	81.3 (73.5–87.2)
P		0.1733	0.4944	0.4350	0.4354	0.2698	0.4099
**Education**							
High school or lower	68	33.8 (23.7–45.7)	26.5 (17.4–38.0)	2.9 (0.8–10.1)	77.9 (66.7–86.6)	38.2 (27.6–50.1)	80.9 (70.0–88.5)
College or higher	126	41.3 (33.1–50.0)	27.0 (20.0–35.3)	2.4 (0.8–6.8)	65.1 (56.4–72.8)	32.5 (25.0–41.1)	84.1 (76.8–89.5)
P		0.1188	0.9386	0.8142	0.0632	0.4260	0.5660
**Occupation**							
Unemployed	48	31.3 (20.0–45.3)	22.9 (13.3–36.5)	6.3 (2.1–16.8)	68.5 (54.7–80.1)	37.5 (25.2–51.6)	81.3 (68.1–89.8)
Employed	146	41.4 (33.4–49.2)	28.1 (21.4–35.9)	1.4 (3.8–4.9)	69.9 (62.0–76.7)	33.6 (26.4–41.6)	83.6 (76.7–88.7)
P		0.2243	0.4834	0.0642	0.8844	0.6186	0.7115

The association between protective antibody levels in cord sera and maternal demographic characteristic was also shown in Table 6. Percentages of newborns with protective anti-Dtx and anti-Ttx IgG were positively associated with their mothers’ age and birth place, and negatively associated with the mothers’ other socio-demographic characteristics including gravidity, parity, birth place, residence area, education or occupation. However, for anti-Ptx IgG, anti-measles, anti-mumps IgG and anti-rubella, there were no statistically significant correlations between protective percentages and all these maternal factors.

## Discussion

The present study revealed that 61.3%, 73.2%, 97.4%, 30.4%, 65.5% and 17.0% of newborns had no protection against Dtx, Ttx, Ptx, measles, mumps and rubella, respectively. Our findings suggested that the pregnant women and newborns were generally lack of protective antibodies to DTP and MMR vaccines, placing them vulnerable to these vaccine-preventable diseases in China. The mothers in this study were born in 1977–1995, most of them could get the antibodies from DTwP and MV vaccination. Mumps and rubella vaccine begin in 1990s, however, the vaccination coverage was low at that time because of self-supported and voluntary type in China^21–23^. The majority of antibodies against mumps or rubella, however, must be naturally acquired.

The percentages of mothers with no protection for Dtx and Ttx were distinguished higher than those in another two previous studies among adults carried in Weifang (none of the studied subjects had no protection for Dtx)^24^ and Netherlands (only 1% of the mothers had no protection for Dtx and Ttx)^1^. The discrepancy could be explained by applied different ELISA kits and cut off values. When this same value (0.01 IU/ml) was adopted, none and 18.6% of the mothers had no protection for Dtx and Ttx. However, we adopted 0.1 IU/ml as the cut off values, according to the manufacturer’s instructions. The percentages of subjects with unprotected anti-Dtx and anti-Ttx level in a recent Turkey study were similar to the present data. That study demonstrated that 65% and 69% of adult had no protection for Dtx and Ttx^25^, which was also evaluated with Euroimmun ELISA kits and the same cut off value. There was no diphtheria case reported in the latest two decades in Beijing, which meant nature boosting by circulating *C. diphtheria* was less occurred^26^. The prevalence of protective anti-Dtx and anti-Ttx IgG were higher in the newborns of mothers aged ≤26y than in those of mothers aged >26y. This discrepancy could be caused by greater proportion of vaccination and less waning immunity in mothers aged ≤26y. The prevalence of protective anti-Dtx (*P* < 0.0001) and anti-Ttx IgG (*P* = 0.0003) were significantly higher in the newborns of mothers born in Beijing than in those of mothers born in other cities. This unexpected significant difference reminded us that the anti-Dtx and anti-Ttx IgG levels had been acquire through DTP vaccination, and the difference in subgroup was attribute to different DTP coverage.

Quite different from diphtheria, the prevalence of protective anti-Ptx in the newborns was negatively associated with mothers’ age, birth place, or other socio-demographic characteristics of the mothers. The anti-Ptx IgG below the lower limit of detection (5 IU/ml) occurred in 136 (70.1%) mothers. The different distribution patterns between anti-Dtx and anti-Ptx IgG induced by one combined vaccine indicated that vaccine-induced pertussis immunity acquired during childhood was not long lasting and insufficient for protecting pregnant women in China. It has been more than 19 years since these pregnant women in our study were vaccinated in childhood. Two pregnant women (1.0%) have anti-Ptx IgG ≥ 100 IU/ml, who should have a real recent pertussis infection. Other seroprevalence studies in pregnant women living in developed countries also revealed that 1–2% of them could have recent pertussis infections^17,27^. It was also in line with the incidence of 1% for recent *B. pertussis* infection estimated in another study on seroprevalence of pertussis among adult in child-bearing ages^28^. These cases have exactly proved that the pregnant population was vulnerable to *B. pertussis* infection. Meanwhile, for 26.3% pregnant women who had anti-Ptx IgG 5–<40 IU/ml, we could not eliminate the possibility of waning immunity by possible expose to pertussis in the past. One mother with pertussis would, no doubt, transmit this infection to their young babies.

We demonstrated that not only low protective level against *B. pertussis*, but also discordance between anti-Ptx and anti-Prn seropositivities. The proportions of mothers with detectable anti-Prn level (48.5%) and anti-Prn ≥100 IU/ml (3.6%) are higher than the corresponding results from anti-Ptx IgG (29.9%, 1.0%). The discordance between anti-Ptx and anti-Prn seropositivities was similar to a study in USA, which demonstrated that detectable cord level of anti-Ptx and anti-Prn IgG were found in 34.7% and 80.2% of samples, respectively^29^. This discordance might be due to cross-reaction for anti-Prn, or different kinetics for waning immunity for these two antigens.

Infants <1 years old have become the most vulnerable population for measles-related complication. The number of cases of measles in infants aged <9 months was high and increases markedly after the infants reach to 6 months^4^, in whom prevention of measles should be considered a priority. The implement of women of child-bearing age vaccination against for MMR vaccine will have benefits for this population. Since the measles contagiosity was calculated to be nearly 100%, a significant reduction of the circulating virus and its transmission cannot be achieved unless the population immunity is 95% and more^30^. The results of our study showed a insufficient protective proportion (70.1%) in pregnant women. Routine MV administration was implemented in early 1980s, the vaccine coverage were gradually raised. However, no difference of maternal anti-measles antibody between 1990s-born women and 1980s-born women (seropositivity rate 65.0% vs. 58.2%; χ^2^ = 0.798, *P* = 0.3716) could indicate that vaccine-induced immunity wane with time. Together with the seroprevalence of DTP vaccine-specific IgG, these results suggested that a lifelong vaccination might be necessary to control these vaccine-preventable diseases.

As well as resurgence of measles, outbreaks of mumps and rubella have also reemerged in some areas and countries^31–33^. In China, the incidence of mumps has declined from 100–1000/100000 in the 1970s to10/100000 in 2001. However, the incidence of mumps has remained at level more than 10/100000 in recent years (12.7655/100000 in 2016 and 18.3166/100000 in 2017). In the last decade, outbreak of mumps still mainly affected children and adolescents <15 years^22^, and sometimes also infected infants <12 months^33^. The protective immunity of these infants was mainly acquired from their mothers. In this study, 70.6% of the mothers were born before 1990 and haven’t get MRV or MMR vaccine inoculation. However, 96.3% (129/134) of them have detectable anti-mumps antibody. The data from mothers born in 1990s were consistent with these finding. As mump vaccination was self-supported and voluntary in 1990s, the coverage rate was low in that time. Most of 1990s-born women 91.7% (55/60), however, had detected anti-mumps antibody. These high percentages of women with protective anti-mumps antibody suggested that mumps virus could widely circulate in China.

The protective rate of anti-rubella among pregnant women was 84.5%, and this means that about 15.5% of them were susceptible to rubella. This positive rate was high, but most of the protective immunity was not induced by immunization, and which suggested that most of the women were infected with this virus in the past. One study suggested that the peak age of rubella cases gradually changed from <15 years to adults in China after 2005^33^. This present data was particularly worrisome, as rubella infection in pregnant women can result in miscarriages, stillbirths, and congenital rubella syndrome (CRS), a constellation of birth defects that often includes cataracts, hearing loss, mental retardation, and congenital heart defects^32^. The high positive rate should be caused by a common exposure to this virus in women, which indicated undoubtedly a threat to the fetus health. However, according to the data of CRS surveillance conducted in some provinces, reported CRS case was scarce in China. The reasonable explanation for this contradiction might be the majority of the women were infected by this virus before pregnancy. Another possibility was that the incidence of CRS was underestimated, as the national rubella and CRS surveillance has not yet been established. Adopting the cut off value of 10 IU/ml^18–20^, our protective prevalence was comparable with the 85.8% seroprevalence reported in southern Italy^34^. However, it was lower than the seroprevalence of 93.3%, 92.8% and 91.6% recorded in Burkina Faso^35^, Haiti^36^, and Saudi Arabia^37^, respectively. The substantial susceptible proportions of childbearing-age women suggest a catch-up campaign against rubella. With the implementation of rubella vaccination strategies, endemic rubella transmission has been interrupted in the Americas since 2009^38^. A partial vaccination strategy results in rubella outbreaks in Japan and elsewhere^39^.

There were certain limitations in this study. One of the limitations of this study was all subjects were collect in only one hospital. An analysis of the comparison of the distribution of samples from the population of Beijing and the whole country is required. Multi-center studies in the whole country should be performed in the future to give evidence for national improvement of immunization strategies. Second, the information on the vaccination status of pregnant women could not be obtained. Third, only ELISA seronegativity was evaluated, which was not complete indicator for missing immunity. It would make the study better that some cell-mediated immunity could be involved. In view of the numerous cases, and the consistency of tested antibody concentration between mother and babies, the present data was an important evidence for promoting the current immunization strategy in China.

The present study estimated the immunity level of mother and their babies against 6 children vaccines. The protective rates varied markedly, ranging from 2.4% (pertussis) to 83.0% (rubella). The majority of newborns were unlikely to be protected from pertussis through maternal antibodies. The protective rates for diphtheria and tetanus were 38.7% and 26.8%, respectively. Only 1.0% newborns had protection against for all three components of DTP. Although many immunization programs could play role in prevent pertussis in infants^40^. Maternal immunization has been demonstrated to be the most cost-effective strategy currently and has been implemented in many countries. Our results support a dose of Tdap booster during pregnancy in China. The protective rates for measles, mumps and rubella were 69.6%, 34.5% and 83.0%, respectively. The protective immunity was not enough to eliminate any of these viruses. To control these diseases in China, preconception or pregnancy screening for MMR should be conducted^41^. Then, vaccination of susceptible women of childbearing age or vaccination in the postpartum period should be strongly implemented. The MMR vaccination in childhood should also be strengthened.

In conclusion, our results showed that most of newborns were susceptible to diphtheria, tetanus, pertussis and mumps, almost one-third of this population had no immune protection against measles, and about one-sixth of them were under threat of rubella infection. The current immunization programmer is not effective enough to control and eliminate DTP and MMR-associated diseases. The results supported a dose of Tdap booster immunization during pregnancy. Preconception screening for MMR immunity should be investigated, and one inoculation could be considered for the susceptible women at childbearing age.

## Materials and Methods

### Maternal and cord serum samples

From June 2016 to March 2017, pregnant women who were about to deliver their baby in the Shunyi Women and Children’s Hospital of Beijing Children’s Hospital could be asked to enroll in this study. The cases with chronic infectious diseases (pulmonary tuberculosis), immune system related diseases, and other chronic medical conditions (diabetes, hypertension, liver, kidney diseases) were excluded. Newborns with premature ruptures of fetal membranes, amniotic fluid infection, premature birth, low birth weight, lower apgar scores were also excluded. A routinely maternal blood sample was obtained at 35 weeks of gestational age, and a cord sample was collected in 20 min after delivery. Information about subject’s age or date of birth, gestational age, birth place (Beijing or other city), residence area (urban or rural), gravidity (one or more), parity (one delivery or more), education (Non-educated/Primary school/Junior high school/Senior high school/College or higher) and occupation (Unemployed/Employed) was also collected. This study was reviewed and approved by the Ethics Committee of Beijing Children’s Hospital Affiliated to Capital Medical University. All methods were performed in accordance with the relevant guidelines and regulations. Written informed consent was obtained from all pregnant women at the time of enrollment for their blood or cord samples to be used for research on maternal/infant infectious diseases.

### Serological testing

Dtx, Ttx, Ptx, Prn, measles, mumps and rubella specific IgG antibodies were determined by using commercially available ELISA kit (Euroimmun, Lübeck, Germany), according to the manufacturer’s instructions. The results were obtained in terms of IU/ml for Dtx, Ttx, Ptx, Prn and rubella, IU/l for measles, and RU/ml for mumps.

### Cut-off values

Results were evaluated with regard to protection levels, seropositivity and booster vaccine indication according to cut-off values, as recommended by the manufacturers and previous studies^17^. These taxonomic rank and corresponding cut-off values were listed as following:

Dtx: <0.01 IU/ml (undetectable), 0.01–<0.1 IU/ml (no protection), 0.1–1.0 IU/ml (short term protection), >1.0–1.5 IU/ml (long term protection/booster after 5 years), >1.5–2.0 IU/ml (long term protection/booster after 7 years) and >2.0 IU/ml (long term protection/booster after 10 years).

Ttx: <0.01 IU/ml (undetectable), 0.01–0.1 IU/ml (no protection), >0.1–0.5 IU/ml (short term protection), >0.5–1.0 IU/ml (long term protection/booster after 3 years), >1.0–5.0 IU/ml (long term protection/booster after 5 years) and>5.0 IU/ml (long term protection/booster after 8 years).

Ptx: <5 IU/ml (undetectable), 5–<40 IU/ml (seronegative), 40–<100 IU/ml (equivocal) and ≥100 IU/ml (indicates an acute infection or recent vaccination/seropositive).

Measles: <50 IU/l (undetectable), 50–<200 IU/l (seronegative), 200–<275 IU/l (equivocal) and ≥275 IU/l (seropositive).

Mumps: <2 RU/ml (undetectable), 2–<16 RU/ml (seronegative), 16–<22 RU/ml (equivocal) and ≥22 RU/ml (seropositive).

Rubella:<1 IU/ml (undetectable), 1–<8 IU/ml (seronegative), 8–<11 IU/ml (equivocal) and ≥11 IU/ml (seropositive).

International assigned protective concentrations were used to assess the percentage of newborns with protective IgG concentrations (anti-measles ≥200 IU/l^18,19^ and anti-rubella ≥10 IU/ml^18–20^). Protective concentrations for anti-Ptx and anti-mumps IgG were not international assigned, but locally assigned cut-off values >40 IU/ml^17^ and ≥45 RU/ml^18,19^, respectively, were used as previously described.

### Data analysis

Maternal age, gestational age and birth weight are described as mean and range. Antibody levels were expressed with 25%, 50% and 75% percentile, respectively. Antibody seroprevalence was calculated with 95% confidence interval (CI). The seroprevalence among different groups was compared with the chi-square test. Data were analyzed using the GraphPad Prism software (version 5; GraphPad Software, La Jolla, CA, USA) and SPSS (version 19.0). *P* ≤ 0.05 was considered statistically significant.
